# Developing a best practice framework for clinical competency education in the traditional East-Asian medicine curriculum

**DOI:** 10.1186/s12909-022-03398-4

**Published:** 2022-05-10

**Authors:** Sang Yun Han, Seung-Hee Lee, Han Chae

**Affiliations:** 1 College of Korean Medicine, Daejeon University, Daejeon, 34520 South Korea; 2 Department of Medical Education, Seoul National University College of Medicine, Seoul, 03080 Korea; 3 School of Korean Medicine, Pusan National University, 49 Busandaehak-ro, Mulgeum-eup, Yangsan-si, Gyeongnam 50612 Korea

**Keywords:** Medical education, Traditional East-Asian medicine, Clinical competency, Best practice framework (BPF), Objective Structured Clinical Examination (OSCE), Clinical performance examination (CPX)

## Abstract

**Background:**

While clinical competency is crucial for traditional East-Asian medical education, available studies on the educational system for fostering clinical performance are scarce. This study aimed to review the educational system, curriculum, facilities, and management of current traditional East-Asian medicine in a well-established university of Korea and develop a Best Practice Framework (BPF) of clinical competency education.

**Methods:**

The clinical competency education system in Pusan National University School of Korean Medicine was systematically described through 5 steps of governance of the educational system, competency of the graduates, educational resources, assessment strategies and tools, and gaps in the curriculum. We also reviewed the experiences in education and the points to be improved.

**Results:**

The Office of Traditional Korean Medicine Education governs the development, implementation, and evaluation of the educational curriculum for cultivating students’ clinical competency. Medical students have undertaken 39 modules of clinical biomedicine and 21 of traditional medicine during the clinical clerkship courses in an affiliated hospital, Clinical Skill Practice Center, clinical research center, practice lab for medical herb, and other locations. After training, 15 modules of simulated clinical training using standardized patients, students’ clinical competency are evaluated by a Clinical Performance Test using a Clinical Performance Examination (CPX) and an Objective Structured Clinical Examination (OSCE) for biomedical and traditional medical skills.

**Conclusions:**

A clinical competency framework is required for a qualified physician of traditional East-Asian medicine. This study reviewed the current well-organized educational system of Korean traditional medicine in detail, which can be used for the BPF of competency-based clinical education. We expect the current study to be a representative reference for establishing an educational system of traditional medicine such as acupuncture and medical herbs in other countries.

## Background

The shift to Competency-based Medical Education (CBME) that emphasizes actual clinical performance has brought about many clinical skills education and evaluation changes. The goal of medical education has been relocated from learning knowledge in classrooms and textbooks, as “what students know”, to acquiring clinical competency in real life as “what they can do” [1]. In Miller’s pyramid [2], which is widely used as a framework for evaluating clinical competence, “shows how” and “does” are superordinate to “knows” and “knows how.” This means that clinical competency should be evaluated by measuring not only knowledge, but also skills and attitudes such as clinical performance and communication [3].

Nowadays, patients want qualified care from well-trained medical professionals with up-to-date medical knowledge and proficient clinical expertise [4, 5]. Therefore, the medical education for prospective clinicians should provide advanced knowledge, efficient skills, and sufficient experience that would lower the risk of medical malpractice and accidents and increase the possibility of precise diagnosis and adequate treatment [5, 6]. In that respect, Objective Structured Clinical Examination (OSCE) and Clinical Performance Examination (CPX) are intensely used in most medical schools to teach and evaluate the clinical competency of medical students.

The OSCE was improvised by Harden and co-authors in 1975 [7, 8], making students perform clinical skills on manikins or standardized patients within an allocated time [9]. Numerous medical schools around the globe have introduced the OSCE to evaluate the clinical competency of their students [10, 11] after its incorporation as clinical skills education by Dundee University of United Kingdom [12]. The CPX evaluates clinical procedures of medical history recordings, physical examinations, differential diagnoses, prescriptions, and patient education using simulated clinical settings and the standardized patient [9]. The standardized patient was developed by Prof. Barrows of the University of California in 1963, and the CPX was implemented for students at the University of Southern Illinois School of Medicine in 1985. The CPX and OSCE, along with the standardized patient, are widely used to evaluate medical practitioners’ medical knowledge, clinical skills, and attitude in medical education [13–16].

As for the nationally recognized qualification examination, the use of OSCE and CPX as clinical competence tests have been expanded. In the United States, these tests were introduced to evaluate foreign medical school graduates in 1998 and comprised the clinical skill test, along with using the standardized patient for United States Medical Licensing Examination (USMLE) step 2 from 2004. Furthermore, the Japanese Kyoyo test for 2 years of clinical training also comprised an OSCE from 2005 [6, 17–19].

In Korea, medical schools of biomedicine have installed clinical competency education and tests using OSCE and CPX for third and fourth graders after finishing core clinical clerkship programs [20] to evaluate academic achievements and secure clinical competency [21].

The OSCE and standardized patients were introduced by Seoul National University College of Medicine in 1994 and 1996, respectively [22, 23], and administered as a major part of the National Licensing Examination of Korean biomedical doctors in 2009 [17, 24]. Subsequently, all 40 Korean medical schools of biomedicine have used OSCE and CPX as pivotal clinical education and evaluation programs to nurture medical students’ clinical competence [21] under the systematized long-term plan since 2009 [6, 20, 25]. Therefore, graduates appraise these educational experiences as beneficial for nurturing clinical proficiency and self-confidence [26, 27]. Additionally, hospital residents consider the experiences of OSCE and CPX as valuable in becoming competent clinical specialists [5].

The clinical competency test as a part of the Korean National Licensing Exam has been introduced for dental doctors from 2021; in upcoming years, it will also be introduced for the doctors of traditional Korean medicine [28]. Likewise, Taiwan established clinical competency tests in the national exam as an imminent goal [29], and China allegedly started clinical competency tests for traditional medicine [17].

The Incorporation of clinical competency tests into the National Licensing Exam represents the implementation of CBME in traditional medicine. Hence, it is important to establish the competencies required to have the knowledge, skills, and attitudes required for a traditional medical doctor and provide an appropriate education system for fostering these. To implement CBME, adequate teaching, assessment strategies, and resources must be prepared, and governance in charge of the overall educational system is needed [30, 31].

The Korean version of clinical competency education and evaluation in traditional East-Asian medicine (Table 1) has been implemented by the Pusan National University (PNU) School of Korean Medicine [5]. After providing the outline (2007) and draft (2008) of clinical skills education as part of the curriculum development, the detailed action plan and textbooks “‘Guidebook for Clinical Skills” [32] and “Guidebook for Clinical Procedures” [33] were finalized in 2010 [17, 32, 34]. The clinical competency education consisted of OSCE, CPX, and standardized patients; the Clinical Performance Test program has been working successfully since 2011, and this system was adopted by three Korean colleges of traditional medicine [35–37].

**Table 1:** The education and evaluation of clinical skills and procedures for clinical competency in traditional East-Asican medicine

Category	Contents (modules or items)	Place	Duration
Education	Clinical skills covering medical examination (26), Clinical tests and questionnaires (12) and clinical techniques (18)	Affiliated traditional medicine hospital (8 clinical departments) Clinical Skill Practice Center	20 weeks
	Processing & preparation of medicinal herbs (4)	Practice Lab for Medical Herb	2 weeks
	Research ethics and clinical research	Affiliated Clinical Research Center	2 weeks
	Simulated Clinical Experience progam using simulated clinical setting and standardized patient (15)	Clinical Skill Simulation Room	1 weeks
Evaluation	Clinical Performance Test (Objective Structured Clinical Examination (6), Clinical Performance Examination (3), Acupuncture point location & placing needles (2) and Preparation & processing of medical herbs (1))	Test rooms Acupuncture Practice Room Practice Lab for Medical Herb	2 days (120 min./person)

Although the clinical use of medical herbs and acupuncture is a subject of great interest worldwide, relevant studies on the efficient and organized educational system for cultivating clinical competency of traditional East-Asian medicine remain scarce [38]. In this context, the present study aims to develop a Best Practice Framework (BPF) of clinical competency education using the case of one traditional Korean medicine school.

The governance of clinical education, steering bodies, facilities, academic calendar, the curriculum, textbooks, educational modules, experiences from the operation, and items to be improved will be shared and discussed.

We hope that this study will provide practical experiences of traditional medicine education to be considered as a reference or guide by countries and colleges looking for the established clinical education.

## Methods

The current study systematically reviewed structured clinical competency education of traditional Korean medicine that has been practiced for a decade in the School of Korean medicine, PNU. The clinical competency education in there includes OSCE and CPX along with ten modules of Problem-Based Learning (PBL), however it was not included in this study for its focusing on the differential diagnosis rather than hands-on skills. The clinical education system, clinical skills and procedure education, and Clinical Performance Test consisting of OSCE and CPX were showed using five steps of BPF.

- Step 1. As for the governance of the educational system, the organizational structure and responsibilities of the Office of Traditional Korean Medicine Education (OTKME) were detailed.
- Step 2. The clinical competency of the graduates as a result of clinical education was described.
- Step 3. Educational resources, including practice rooms and guidebooks, were reviewed. The utilization of practice rooms such as the Clinical Skill Practice Center (CSPC), the Clinical Skill Simulation Room (CSSR), the Acupuncture Practice Room (APR), and the Practice Lab for Medical Herbs (PLMH) was also presented. The contents of the clinical education were reviewed in two perspectives of clinical skills and procedures, which were detailed in the “Guidebook for Clinical Skills” [32] and the “Guidebook for Clinical Procedures” [33], respectively. A comprehensive review was provided on the educational curriculum and learning issues such as clinical skills, an academic calendar, instructors, an educational curriculum, and standardized patients for clinical procedures.
- Step 4. Assessment strategies and tools used for evaluating students’ clinical competency in traditional Korean medicine were illustrated. The agenda, governance, question items, and evaluation criteria of the Clinical Performance Test (CPT) that inspect the proficiency of clinical skills and procedures using OSCE and CPX were presented. Furthermore, the detailed processes for testing clinical competency related to acupuncture needling and the processing of medical herbs were also provided, considering their importance for the education of traditional medicine.
- Step 5. Gaps in the clinical curriculum to be improved were described.

## Results

### Governance of the educational system

The OTKME is the central body of development and evaluation of the curriculum, research on teaching-learning methods, management of human resources, equipment and consumables, supervising the educational curriculum for clinical education (Table 1), and others. The OTKME is also in charge of publishing the textbooks “Guidebook for Clinical Skills” [32] and “Guidebook for Clinical Procedures” [33], training and utilizing standardized patients, and supervising the use of the CSPC [39], the Acupuncture Practice Room (APR) and the Medical Herbs Practice Room. They organize and operate the CPT, support the PBL classes, manage the web server for Computer-based Tests (CBT), and receive feedback from students on the curriculum and other items.

The OTKME is controlled by a chairperson and three full-time officers and has two steering bodies; the Steering Committee of OTKME, which discusses curriculum, budget, admission, and graduation issues, and the Steering Committee of Research Program, which supervises the students’ research programs. There also are two committees, including the Committee for Teaching-Learning Method Development and the Committee for Curriculum Assessment for improving the quality of traditional medical education in general.

### Clinical competencies of the graduates

There are five core competencies designated for the clinical education of traditional Korean medicine as follows.

1. The procedures and methods for keeping the clinical setting clean and sterile.
2. The accurate practice of physical examination on patients.
3. The correct administration of traditional medical diagnosis and appropriate explanation of its results to the patients.
4. Safe and effective performance of acupuncture, moxibustion, cupping, and pharmacopuncture.
5. Proficient preparation, processing, and prescription of medical herbs.

### Educational resources

**Fig. 1:**
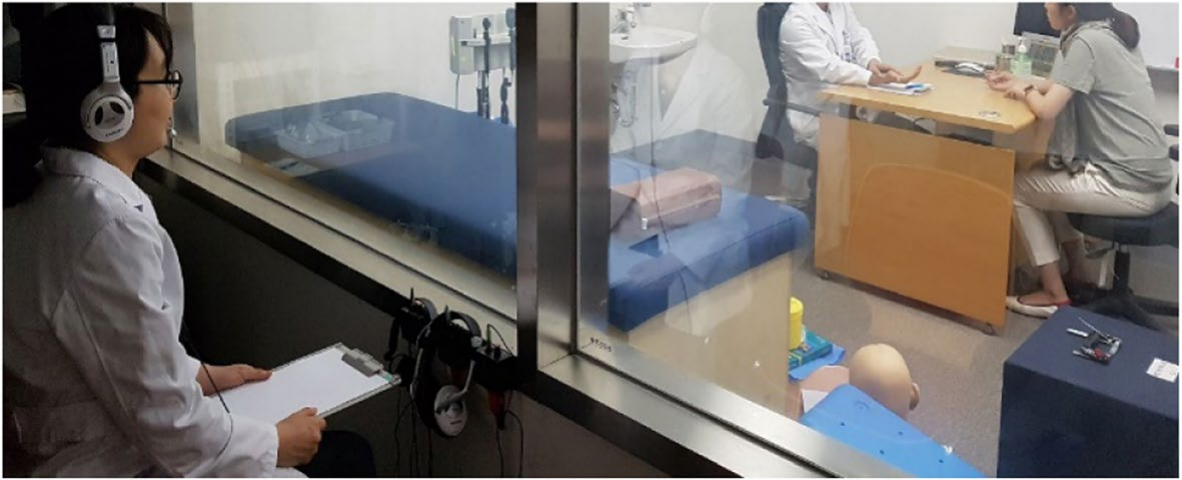
Clinical Skill Simulation Room for Simulated Clinical Experience program. Student is watching and hearing the conversion between standardized patient and other student for feedback

The CSPC, with a space of 118.29m^2^, has 17 kinds of human manikins and body models, beds for Chuna Manual Therapy, inspection devices [39] of ultrasound, electrocardiogram, ryodoraku, and more for teaching clinical skills and procedures. CSPC also has two CSSR with a one-way mirror and *closed-circuit recording system*, which are equipped with devices for teaching procedures of abdominal examination or physical examinations using standardized patients (Fig. 1). It also has consumables, e.g., tongue depressors, cotton swabs, hand sanitizer, disinfected gloves, acupuncture needles and more, for learning and practicing clinical skills and procedures free of charge.

The APR has nine beds and several types of acupuncture needles and moxibustions to teach needling practice to students. The APR is equipped with a video recording system and big-screen TVs to enlarge the instructors’ needling techniques and procedures. The PLMH allows students to examine the texture, taste, and smell of textbook-listed medical herbs and also has electric heating and boiling appliances, a distributor, a freeze dryer, a pill-making machine, and other items for practicing processing and boiling techniques.

There is a “Guidebook for Clinical Skills” for efficient implementation and management of clinical education, revised annually by the academic staffs. The Guidebook has 4 chapters of medical examination (26 modules), clinical tests and questionnaires (12 modules), clinical techniques (18 modules), and medical herbs (4 modules). In other words, it provides 60 modules of clinical skills (Table 2) with preparation, explanation of skills, references, supplementary checklists, and scoring criteria for self-directed preparation of clinical proficiency tests or CPT [32].

**Table 2:** Contents of 60 OSCE modules covering biomedicine and traditional medicine for students of traditional East-Asian medicine

Category (modules)	Biomedicine (39 modules)	Traditional medicine (21 modules)
Medical examination (26)		
General (12)	1. Blood pressure check, 2. Fundoscopy, 3. Otoscopy, 4. Examination of nose, 5. Examination of neck, 6. Examination of lungs, 7. Examination of heart, 8. Examination of abdomen, 9. Examination of anus and rectum	1. Pulse diagnosis 2. Tongue diagnosis 3. Abdominal examination
Nervous system (5)	1. Examination of cranial nerves, 2. Examination of motor function, 3. Examination of sensory function, 4. Examination of reflexes, 5. Examination of cerebellar function	
Musculoskeletal system (4)	1. Physical examination of cervical vertebrae, 2. Physical examination of lumbar vertebrae, 3. Physical examination of shoulder joint, 4. Physical examination of knee joint	
Obstetrics/Gynecology (5)	1. Examination of breast, 2. Vaginal smear test, 3. Examination before delivery, 4. Visual inspection, palpation, and combined examination on genital area, 5. Vaginal sonography	
Clinical tests and questionnaires (12)		
Internal medicine (6)	1. Presentation of chest X-ray, 2. Electrocardiographic examination, 3. Pulmonary function test, 4. Abdominal sonography	1. Ryodoraku diagnosis, 2. Heart rate variability
Neuropsychiatry (2)	1. Korean-Mini Mental State Examination	1. Diagnostic interview for Hwa-Byung
Sasang medicine (3)		1. Questionnaire for the Sasang Constitution Classification II, 2. Facial Measuring using 3D Facial Analysis Machine, 3. Anthropometric measurement
Pediatrics (1)	1. Scoring Atopic Dermatitis in children	
Clinical techniques (18)		
General (14)	1. Intravenous blood sampling, 2. Male catheter insertion, 3. Female catheter insertion, 4. Wound dressing, 5. Burn dressing, 6. Incision and drainage of abscess, 7. Suturing	1. Filiform acupuncture needle, 2. Moxibustion treatment, 3. Cupping therapy, 4. Pharmacopuncture, 5. Needle-embedding therapy, 6. Chuna manual therapy on Posterior Inferior Ilium Deviation, 7. Interferential Current Therapy and Ultra sound treatment for low back pain
Emergency (4)	1. Adult basic life support - Cardio-Pulmonary Resuscitation and Automated External Defibrillator, 2. Airway obstruction by foreign body (baby), 3. Splinting of extremities	1. Febrile convulsions (children)
Medical herb (4)		
Preparation & processing of medical herbs (4)		1. Stir-baking of herbals, 2. Boiling methods for herbal decoction, 3. Concentrating of extracts and making granule with freeze-drying, 4. Preparation and storage of pharmacopuncture solution

### Development of clinical competencies in the clinical curriculum

#### Teaching and learning strategies

The evidence-based teaching on traditional medicine through the development of curriculum, class module, and teaching methods has been implemented in Korea [40–44], and the educational system of biomedicine with proven efficacy, e.g., integrated block lecture in clinical courses, PBL, OSCE and CPX, modified to include clinical modalities of traditional East-Asian medicine were adopted in PNU. Furthermore, issue-centered group debate programs, replication of previous clinical studies, and introductive courses using animation characters [45] have been developed to increase the quality and efficacy of traditional medicine education [5, 40, 46].

After completing the curriculum for medical theories and knowledge with didactic classes, a total of 24 (20 + 2 + 2) weeks of clinical education in affiliated hospitals are prepared for the clinical clerkship program and education of clinical skills and procedures (Table 1). The clinical skills and procedures education are pivotal for efficient training of medical professionals that would have effective and safe diagnosis and treatments, professional and humanistic communication and relationship with patients, decreased incidence of medical accidents, and maximized patient satisfaction [47, 48]. These are required as crucial competencies of primary care physicians, and medical students of traditional Korean medicine are learning clinical skills and procedures of both traditional medicine and biomedicine.

Ten weeks are allocated to the later part of the second semester of third grade and 14 weeks to the earlier part of the first semester of fourth grade. Twenty weeks are allocated for 56 clinical modules (Table 2), case reviews, and clinical observations on major diseases in 8 clinical departments of internal medicine, acupuncture and moxibustion, ear, nose and throat and dermatology, pediatrics, obstetrics, and gynecology, Sasang constitutional medicine, rehabilitation and neuropsychiatry at an affiliated traditional Korean medicine hospital. Two weeks are assigned for 4 clinical modules (Table 2) related to the processing of medical herbs, making a decoction, boiling, freeze-drying and manufacturing pills, and preparing pharmacopuncture solutions. Finally, 2 weeks are used for the research ethics and clinical research design at the affiliated clinical research center.

Clinical skills education is usually implemented by hospital staffs and residents at CSPC and/or an affiliated hospital. For example, clinical staff of the rehabilitation department at the affiliated hospital teach Chuna manual technique for posterior inferior ilium deviation during their lecture on physical examination of cervical and lumbar vertebrae. Generally, 4 h of class time are allocated for each clinical technique, and group or individual practice after school might be done at CSPC.

Sixty modules of clinical skills (Table 2) described in the guidebooks cover biomedicine (39 modules) and traditional medicine (21 modules) altogether. Thirty-nine biomedicine modules (Table 2) can be categorized into the medical examination (23 modules), clinical tests (6 modules), clinical techniques (10 modules), and 21 traditional medicine modules. (Table 2) includes medical examination (3 modules), clinical tests (6 modules), clinical techniques (8 modules), and medical herbs (4 modules).

There are clinical skill modules for traditional medicine, e.g., pulse diagnosis, tongue diagnosis, acupuncture needling, moxibustion, making and boiling herbal decoction, and preparing pharmacopuncture solution (Table 2), which might represent the characteristic features of traditional medical education. These are newly developed to achieve efficient and structured clinical education as biomedicine does.

The pulse diagnosis module makes the students learn the appropriate posture of body and position of fingers, and the tongue diagnosis module teaches shape and color of the tongue, presence of the white coat, degree of wetness as patterns, and profiles as used in clinics. The education of acupuncture techniques is provided by the department of acupuncture and moxibustion as five modules of filiform acupuncture needling, moxibustion treatment, cupping therapy, pharmacopuncture, and needle-embedding therapy (Table 2). These modules refer to previous didactic lectures on Clean Needle Technique [49], location and characteristics of acupuncture points, acupuncture therapies, and needling techniques. Unlike other clinical skill modules, four modules on medical herbs (Table 2) are provided by academic staffs of herbology and pharmacognosy for 2 weeks in PLMH. These are crucial techniques required for primary care focused on local clinic management [32].

Although the department of acupuncture and moxibustion provides training in treating effects of cerebrovascular disease, spinal and joint problems, and facial paralysis that requires specialized needling techniques, the acupuncture treatments of major diseases are provided by eight clinical departments during the 20-week clinical clerkship (Table 1). Medical students are required to observe the clinical practices of academic staffs and exercise clinical skills of acupuncture and moxibustion by themselves to be proficient [5, 50].

During the 24 weeks of clinical skill education, the medical students are trained to be proficient in clinical skills and exercise by themselves to be prepared for CPT using the “Guidebook for Clinical Skills” as a reference and manual. The OTKME might assign time slots for using CSPC, APR, and PLMH as a team or individually for the students’ afterschool practice.

#### Simulated clinical experience program with standardized patients

After completing 24 weeks of clinical skill education, 1 week of the Simulated Clinical Experience (SCE) program (Fig. 1) using simulated clinical setting and standardized patient provides an opportunity for medical students to learn and improve clinical procedures (Table 1). Standardized patients can provide simulated clinical situations with the desired level of difficulty at any time and any place. In addition, they also may express different types of physical and emotional responses to questions and attitudes of medical students, as well as provide objective feedback on students’ clinical proficiency [51, 52].

The SCE program consists of 15 modules, including dizziness, coughing, insomnia, low back pain, and more (Table 3), easily found in primary care clinics. In addition, the “Guidebook for Clinical Performance” also has corresponding 15 chapters containing history taking, physical examination procedures, differential diagnosis, syndrome identification, patient education, schemas for problem-solving, supplementary information, and references for each module [32].

**Table 3:** Contents of 15 CPX modules educated during the Simulated Clinical Experience program

Department (modules)	Title of scenarios
Internal Medicine (3)	1. I feel dizzy, 2. I have a cough, 3. I have constipation
Otolaryngology (1)	1. I have a runny nose
Sasang Medicine (2)	1. My heart is pounding, 2. Something stuck in my throat
Neuropsychiatry (3)	1. I have insomnia, 2. I am nervous, 3. I cannot remember
Musculoskeletal (2)	1. I have back pain, 2. I have swollen and painful joints
Gynecology (2)	1. I have increased vaginal discharge, 2. Absence of menstrual bleeding
Pediatrics (2)	1. Bed-wetting in kids, 2. My child seems to be developing late

Ten minutes at the CSSR are assigned to the SCE modules with the standardized patient, and students would have 6 occurrences of 15 SCE modules. The students would receive multidimensional feedback on their clinical performance from fellow students (peer perspective), standardized patients (doctor-patient perspective), and academic staffs (teaching/learning perspective), as well as practice by themselves to prepare for the CPT on 15 CSE modules with the help of the guidebook.

The standardized patients of the current SCE program are trained personnel who act as patients during the 15-h scenario training by OTKME; they can also participate in educational programs of other medical schools.

### Assessment strategy and tools

#### Clinical performance test

The purpose of the two-day CPT program is to evaluate the clinical competency of medical students in clinical skills, techniques, and procedures after 25 weeks of clinical education (Table 1) which has 60 modules of clinical skills (Table 2) and 15 modules of clinical procedures (Table 3). If a student scores below 60 in CPT, he or she must take and pass the test next year.

One set of the test (Table 4) for six student examinees is composed of CPX (3 items, 10 min/item), OSCE (6 items, 5 min/item), acupuncture point location, and placing needles (2 items, 7 min/item), and preparation and processing of medical herbs (1 item, 10 min/item); it takes a total of 120 min including waiting time for each examinee.

**Table 4:** Outlined schedule of Clinical Performance Test

Contents (test items per examinee)	Time (min)	Test site
CPX (3) & OSCE (6)		
CPX #1	10	Room 1
OSCE #1	5	Room 2
OSCE #2	5	Room 3
CPX #2	10	Room 4
OSCE #3	5	Room 5
OSCE #4	5	Room 6
CPX #3	10	Room 7
OSCE #5	5	Room 8
OSCE #6	5	Room 9
Acupuncture point location & acupuncture needling (2)	14	Acupuncture Practice Room
Processing & preparation of medical herbs (1)	10	Practice Lab for Medical Herb

There are 10 test rooms with an assigned academic staff for evaluation and observation from other medical schools; the test questions are posted on the doors to be checked by students before entering the room. The examinees may use pencils only and wear their name tags on their chests.

The examinee and examiner may request to stop the test when an emergency occurs, after which the process of the CPT program is paused until the issue or situation is resolved. Examinees with physical or mental disabilities would be assigned at the end of the CPT, and 50% more time would be provided for each test item.

The evaluation criteria and checklists for the CPT program were previously distributed as included in the “Guidebook for Clinical Skills” and the “Guidebook for Clinical Performance,” and the student examinees can prepare for the CPT program on their own with sufficient time. The range of points assigned for each CPT test item is 10 to 24 depending on their difficulty and complexity, and the evaluator scores 0–2 points to each predefined evaluation checklist element.

As for the evaluation checklist of the filiform acupuncture needling module, all processes are to be checked. These include explaining the treatment scheme to the patients, selecting proper acupuncture points for a specific symptom, Clean Needle Technique, placing the needle on acupuncture points, ensuring appropriate posture during the needling, manipulation techniques of reinforcing and reducing for getting deqi, and adequate disposal of used needles, as described in the guidebooks.

#### Clinical performance examination and objective structured clinical examination

**Fig. 2:**
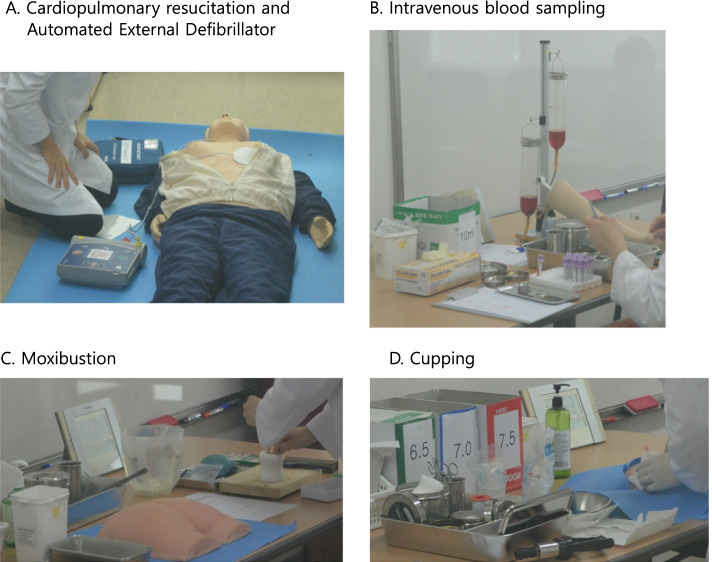
Evaluation of clinical skills using Objective Structured Clinical Examination during the Clinical Performance Test. **A** Cardiopulmonary resucitation and Automated External Defibrillator. **B** Intravenous blood sampling. **C** Moxibustion. **D** Cupping

**Fig. 3:**
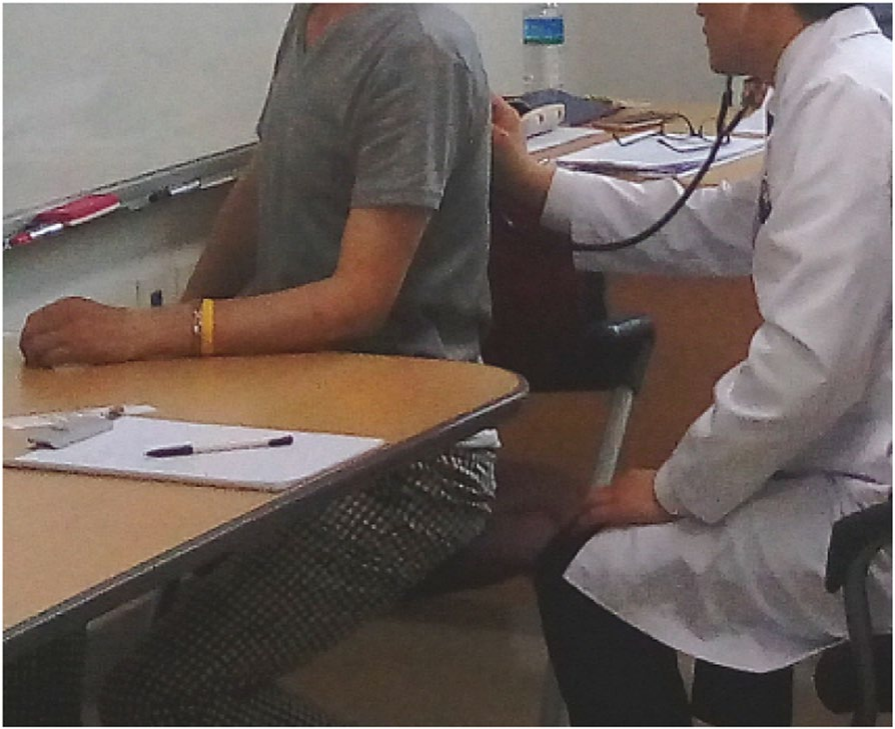
Auscultation using standardized patients during clinical procedure evaluation in Clinical Performance Test

The current CPT program uses six randomly selected items from 60 OSCE modules (Fig. 2) and three items from 15 CPX modules (Fig. 3) trained during clinical education. The CPX and OSCE were incorporated to substantiate the clinical competency of primary care practitioners in medical history taking, physical and clinical examination, clinical reasoning and diagnosis, and effective communication with patients.

The student examinee checks the clinical problem of the standardized patient or the specific clinical skill posted on the door of each test room (1 min before entering) and presents their required clinical competency (Fig. 2) using prearranged devices and consumables. There is the preparation alarm 2 min before the closing alarm which makes the examinee move to the next test room. The examinee can only leave the assigned test room after the closing alarm regardless of whether they have already completed the presentation.

Regarding the CPX, the examinee should obtain clinical information using a physical examination of biomedicine (Fig. 3) and/or four diagnostic methods of traditional medicine, make a correct diagnosis based on acquired clinical evidence, and provide the necessary explanation of the diagnosis to the patients.

As for the clinical information, the student examinee should perform the required number of examinations and get predefined clinical evidence for the standardized patient, which would be used for the scoring. However, as for physical examinations of biomedicine not available with a standardized patient, e.g., digital rectal examination and gynecological bimanual examination, the standardized patient hands over papers with test results when the examinee explains the diagnosis to the patient. Furthermore, the clinical information from tongue and pulse diagnoses of traditional East-Asian medicine will be given accordingly by the standardized patient when the examinee properly follows the clinical procedure.

**Fig. 4:**
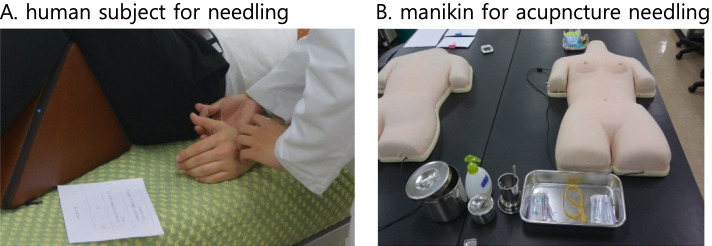
Evaluating acupuncture point location & placing needle during Clinical Performance Test. **A** human subject for needling. **B** manikin for acupncture needling

The clinical skills of traditional medicine comprising acupuncture needling (Fig. 4) and medical herb handling are examined separately after completing the CPX and OSCE, as discussed in the next chapters.

#### Assessment of competence in acupuncture

Six student examinees are grouped into three same-sex subgroups, and each subgroup takes two assignments to attest their clinical competency in point location and acupuncture needling. A total of 15 min is allocated for the test in APR; 7 min would be used for each assignment, and 1 min for changing roles of the practitioner and patient between subgroup members (Table 4). The examinees use their subgroup members (Fig. 4-A) as patients for needling or a manikin (Fig. 4-B) in case of private location or possible bruising, e.g., perineum (CV1), abdomen (CV4), and face (ST2).

The academic staffs of the acupuncture department score the clinical skills, including point location, depth and angle of the needling, manipulation techniques, disposal of used needles, and the Clean Needle Technique [49]. The assignments cover diverse areas, including standard treatment for common diseases, clinical characteristics of major points, and combination of points in Sa-am Acupuncture and Tae-Geuk Acupuncture, along with the modules listed in the “Guidebook for Clinical Skills.”

#### Assessment of competence in processing and preparation of medical herbs

Six student examinees are grouped into two subgroups; one subgroup takes a test to examine clinical competency in processing medical herbs and making decoctions, while the other subgroup waits outside. Each examinee would perform one of three assignments selected from four modules listed in the “Guidebook for Clinical Skills” during 10 min in the PLMH as shown in Table 4.

Academic staffs of herbology and pharmacognosy department evaluate examinees’ clinical knowledge and techniques. These include hand washing and wearing gloves for personal hygiene, safety rules to prevent accidents, cleaning of raw herbs and prevention of contamination, procedures for modifying clinical properties of medical herbs, as well as the use of appliances and devices, including boiling pots, the freeze-dryer, and the powder-maker.

### Gaps in the curriculum

There are several gaps in the current clinical curriculum of traditional medicine. The lack of skill modules to be used in the clinical education of traditional medicine and the need to develop other CPX scenarios and modules for diseases commonly encountered in clinics should be recognized. Furthermore, the acquisition of clinical skills is crucial for students’ clinical competency, but patient-physician interaction (PPI) also requires sufficient time for practice. It is important not only to maintain the difficulty level of each station but also for the evaluators to evaluate the same student’s performance consistently. Finally, oral, informal, formal, descriptive, evaluative, peer, and self-assessed feedback and the currently written one should be considered for the quality improvement of clinical education.

## Discussion

In recent years, the epicenter of medical education has shifted from memorizing medical knowledge to fostering clinical competency and proficiency [21], and the education of traditional East-Asian medicine is vigorously trying to incorporate this trend [39]. Education of traditional Korean medicine is currently considering a transfer from paper-based tests on memorized knowledge to smart device-based multidimensional tests on clinical competency [53]. The inclusion of clinical skills and procedures assessment in the National Licensing Examination for traditional medicine is actively considered [54, 55]. Highly qualified medical professionals with the desired knowledge, skills, and attitudes are a particularly great concern of traditional East-Asian medicine, and experiences with elaborated medical education of traditional Korean medicine are valuable at this point [5, 17, 56].

In this context, we reviewed the clinical competency education system implemented at PNU using BPF in five steps. As for the governance, although the role of the OTKME was satisfactory, the absence of experts in the OTKME should be improved. For better CBME, experts qualified with research experience and research methodology for evidence-based teaching in medical education must be placed at OTKME.

This study systematically reviewed 24 weeks of clinical skills education with clinical clerkship, 1 week of focused training using simulated clinical setting, and a two-day CPT program for evaluating comprehensive clinical competency and proficiency in traditional Korean medicine education implemented a decade ago (Table 1). There were 75 essential clinical competencies covering biomedicine and traditional medicine, required for primary care traditional medical practitioners, and these were standardized as an academic module for clinical education (Tables 2 and 3). Furthermore, it was also revealed that the teaching and evaluation using OSCE and CPX were successfully installed for traditional medicine education [36, 57] with high satisfaction (4.2 and 4.3 out of 5) of medical students [17, 56], and other colleges of Korean medicine are now considering these as a reference.

Other colleges of traditional Korean medicine, unlike PNU with full establishments, are actively enhancing the education system, facilities, curriculum, and staff to improve clinical competency. The OSCE module is being developed by each clinical society, while the CPX module is being developed individually by each university. Eleven out of 12 Korean medical schools implement CPX, and six universities use standardized patients [37]. Wonkwang University launched the Center for Clinical Skills (2014), and provides clinical training programs, e.g., writing medical charts, medical examinations using the virtual patient, and point location and acupuncture needling; this is accomplished during the hospital clerkship period which is limited to 3 months. Daejeon University has the Clinical Center for Clinical Skills and the Guidebook for Clinical Skills and simulated clinical training [35], but the CPT is not established. Wooseok University uses the Center for Clinical Skills (2014) for clinical skills education and evaluation; however, the education and evaluation on simulated clinical training is implemented only for OSCE [36].

The importance of clinical competency has been emphasized likewise in other East-Asian countries and nations with traditional medicine nationally recognized and included as public health, e.g., China, Taiwan and Japan have been encouraging clinical skills education in their traditional medicine.

China allegedly introduced the clinical skills test to the National Licensing Examination for traditional Chinese medicine in 1999, and several universities were reported to have clinical education using standardized patients since 2004 [17]. The examination was designed to evaluate the clinical competency of medical professionals in five categories of differential diagnosis, acupuncture, cupping, tuina (Chuna), and clinical examination [28]. However, detailed reports on clinical skills and procedures education in traditional Chinese medicine universities are not available for review.

Furthermore, Taiwan introduced OSCE to the Professional and Technical Exam for biomedicine doctors in 2013 and considered the same for traditional Chinese medicine. Taiwan aspires to be the first nation to introduce OSCE to the qualification exam of traditional medicine, even though only medical knowledge is appraised at present [29]. The educational universities are using their own evaluation criteria to evaluate students’ clinical competency in traditional Chinese medicine.

As for Japan, the doctor of biomedicine can prescribe herbal medicine even with limited education on traditional Kampo medicine; 90% of medical schools have introductive courses with an average duration of 16.2 ± 8.8 h that might be compared to the 80 h of traditional Chinese medicine courses at Chinese biomedicine universities. Therefore, official clinical competency education for traditional medicine is not provided during the medical school years, and clinical proficiency may be acquired after graduation [58].

This study provided an educational program for competency-based clinical education, which would be the first step in developing a BPF, and there might be a plausible model for traditional medicine by exploring the stakeholder’s perspective and educational outcomes. This has not been achievable in the traditional medicine of other countries; however, with the experience in Korea, patients’ health might be improvement substantially.

Traditional Korean medicine is now preparing transition to competency- and outcome-based medical education [48]. It is about to apply the new standard of a Korean medicine education evaluation and certification system called KAS2021, which strengthens clinical competency [59]. Moreover, the National Licensing Examination of traditional medicine will implement clinical competency tests in the coming years. The clinical education of traditional Korean medicine systematically presented in the current study could serve as a valuable example to be considered as a reference for nations planning to inaugurate or refurbish their educational system of traditional medicine.

Considering the present status of traditional medicine, the clinical competency education program reviewed in the current study has worth establishing organized clinical competency education for East-Asian traditional medicine. However, there are also issues to be tackled for improving the quality of clinical competency education and filling the gaps.

First, the development of refined educational modules for diverse traditional medical modalities is required. The module for pulse diagnosis in the current study consists of simple checks on posture of body, the position of the finger, and simple rhythm of pulse [32], and should be upgraded to include the syndrome differentiation with pulsation using fingertips of student examinees. It would require considerable resources to develop and implement sophisticated and accurate instruments mimicking the human pulse wave [60–62]. In addition, clinical skills modules for unique Korean clinical modalities of Sa-am acupuncture [63] and Tae-Geuk acupuncture [64] would enhance the clinical competency of future medical professionals.

Second, more modules for clinical procedures are required for the clinical competency of students as primary care physicians [34]. Clinical procedure modules for the traditional Korean Sasang typology using objective measures of the Questionnaire for Sasang Constitution Classification, Sasang Personality Questionnaire [65], Sasang Digestive Function Inventory [66], and Sasang Urination Defecation Inventory [67] would be beneficial for the clinicians. Likewise, clinical procedures with medical herbs and acupuncture for frequent diseases of primary care clinics would be needed [68].

Furthermore, since the student examinees focused only on the required activities and easily ignored the proper attitude toward the standardized patient (Fig. 3), the emphasis on the PPI along with clinical skills should be placed throughout education and evaluation [69].

Third, as for managing the CPT program, a training program for evaluators to increase inter-rater reliability is strongly required. The scoring of verbal communication, interview and patient teaching need skilled and experienced attention, unlike physical examination with distinctive evaluation criteria [4]. At present, 5 and 10 min are uniformly (Table 4) allocated to OSCE and CPX modules, respectively, regardless of subject, complexity, and difficulty, and a more efficient and organized evaluation program might be achieved by rearranging test items and redistributing time allocation.

Fourth, retraining clinical procedures using more direct and objective feedback is required. The three types of feedback students receive in the SCE program enable them to evaluate their own clinical performance objectively. However, written feedback can be difficult to immediately improve clinical performance. More sophisticated corrections can be made if the professor and fellow students provide feedback while watching the student’s clinical performance video in a place dedicated to feedback, such as a debriefing room.

Last but not least, a contingency plan for sudden incidents and detailed preparation for disabled examinees should be prepared. The Korea Health Personnel Licensing Examination Institute, which currently governs clinical competency examination for biomedicine and would do the same for traditional medicine, provides an extension of time allocation and assistants during the clinical skills test in accordance with their guidelines for accommodation to examinee with disabilities [70]. In our experience, there was a case in 2016 when a student examinee with prosthetic hand on one arm successfully completed the clinical skills test of point location and acupuncture needling with the help of assistant provided by OTKME.

## Conclusion

The current study aimed at reviewing governance of the educational system, competency of the graduates, educational resources, assessment strategies and tools, and gaps in the curriculum in the established School of Korean Medicine, using BPF of clinical competency education which has not been reported previously. This study will be a reference or guide for other countries and colleges considering restoring and upgrading the clinical education system of their native traditional medicine.

## Data Availability

The datasets used during the current study available from the corresponding author on reasonable request.

## Abbreviations

BPF: Best Practice Framework
CBME: Competency-based medical education
OSCE: Objective Structured Clinical Examination
CPX: Clinical Performance Examination
USMLE: United States Medical Licensing Examination
PNU: Pusan National University
PBL: Problem-Based Learning
OTKME: Office of Traditional Korean Medicine Education
CSPC: Clinical Skill Practice Center
CSSR: Clinical Skill Simulation Room
APR: Acupuncture Practice Room
PLMH: Practice Lab for Medical Herbs
CPT: Clinical Performance Test
CBT: Computer Based Test
SCE: Simulated Clinical Experience
PPI: Patient-physician interaction
